# Brain structure evolution in a basal vertebrate clade: evidence from phylogenetic comparative analysis of cichlid fishes

**DOI:** 10.1186/1471-2148-9-238

**Published:** 2009-09-21

**Authors:** Alejandro Gonzalez-Voyer, Svante Winberg, Niclas Kolm

**Affiliations:** 1 Animal Ecology, Department of Evolutionary Biology, Evolutionary Biology Centre, Uppsala University, Norbyvägen 18 D, 75236 Uppsala, Sweden; 2 Department of Neuroscience, Physiology Unit, Biomedical Centre, Uppsala University, Box 572, 75123 Uppsala, Sweden

## Abstract

**Background:**

The vertebrate brain is composed of several interconnected, functionally distinct structures and much debate has surrounded the basic question of how these structures evolve. On the one hand, according to the 'mosaic evolution hypothesis', because of the elevated metabolic cost of brain tissue, selection is expected to target specific structures mediating the cognitive abilities which are being favored. On the other hand, the 'concerted evolution hypothesis' argues that developmental constraints limit such mosaic evolution and instead the size of the entire brain varies in response to selection on any of its constituent parts. To date, analyses of these hypotheses of brain evolution have been limited to mammals and birds; excluding Actinopterygii, the basal and most diverse class of vertebrates. Using a combination of recently developed phylogenetic multivariate allometry analyses and comparative methods that can identify distinct rates of evolution, even in highly correlated traits, we studied brain structure evolution in a highly variable clade of ray-finned fishes; the Tanganyikan cichlids.

**Results:**

Total brain size explained 86% of the variance in brain structure volume in cichlids, a lower proportion than what has previously been reported for mammals. Brain structures showed variation in pair-wise allometry suggesting some degree of independence in evolutionary changes in size. This result is supported by variation among structures on the strength of their loadings on the principal size axis of the allometric analysis. The rate of evolution analyses generally supported the results of the multivariate allometry analyses, showing variation among several structures in their evolutionary patterns. The olfactory bulbs and hypothalamus were found to evolve faster than other structures while the dorsal medulla presented the slowest evolutionary rate.

**Conclusion:**

Our results favor a mosaic model of brain evolution, as certain structures are evolving in a modular fashion, with a small but non-negligible influence of concerted evolution in cichlid fishes. Interestingly, one of the structures presenting distinct evolutionary patterns within cichlids, the olfactory bulbs, has also been shown to evolve differently from other structures in mammals. Hence, our results for a basal vertebrate clade also point towards a conserved developmental plan for all vertebrates.

## Background

The vertebrate brain is divided into several functionally distinct, albeit interconnected, structures [1-3]. Although specific roles cannot be exclusively attributed to particular structures there is a general consensus that different types of cognitive information are indeed mostly processed within certain brain structures [2,4], and that increased demand on cognitive abilities tends to be associated with an increase in size of the structure processing the information [5-8]. Thus, because of the high metabolic costs of brain tissue [9,10] selection may be expected to target enlargement of only specific structures associated with the behavior or cognitive ability being favored, resulting in brains evolving in a mosaic fashion where changes in the size of specific structures are independent of changes in other structures [1,11,12]. However, it has been suggested that developmental constraints may limit the degree to which structures can evolve independently and that part or whole size dissociations may be inherently less feasible responses to selection than concerted evolution of all structures [13,14]. Under such a concerted evolution model, larger brains would basically be 'scaled-up' versions of smaller brains with conserved relative proportions of their constituent parts, and thus the size of the whole brain is predicted to vary in response to selection on any of its constituent parts [12-14]. Interestingly, initial support for either hypothesis is derived from independent analyses of essentially the same data: brain and structure sizes from four clades of mammals (insectivores, prosimians, simians and bats) [1,13,14]. A subsequent study incremented the coverage of the mammalian database adding 29 more species from 5 orders to the original dataset, and its results appear to support a predominant role for concerted evolution [15]. The only study to have looked beyond mammals supports a predominant role for mosaic evolution of brain structures in birds [12].

The two hypotheses are not presented as alternative or mutually exclusive explanations of brain evolution, and the debate, even among their proponents, has mostly centered on the relative importance of developmental constraints versus adaptive flexibility [1,2,14]. Indeed, not all interspecific variation in structure size within the mammalian brain can be explained purely by developmental constraints as some structures, such as the olfactory bulbs and the limbic system (the hypothalamus, the hippocampus, and the amygdala), do not fit such a model and in these cases mosaic evolution may play a more important role [13,14]. On the other hand, brain evolution is unlikely to be purely mosaic as functionally linked brain structures in both primates and birds do show signals of evolving in a correlated fashion. Moreover, patterns of correlated evolution among structures can be predicted from the degree of anatomical connectivity [3,12]. So, why all the fuss? The influence of developmental constraints relative to flexible evolution of particular structures in response to selection is of foremost importance when investigating the selective pressures which may have influenced observed differences, and as such, to our understanding of brain evolution. In order to identify the units within the brain upon which selection can act it is first necessary to understand the mechanisms behind brain evolution. For example, is larger neocortex size in simians the result of selection pressures for cortically based functions or is it the result of selection acting on a whole other level, for example on non-cortical functions such as motor control via the basal ganglia [14], which resulted in increased brain size and, because of developmental constraints, an enlarged neocortex? In other words, are advanced cognitive traits of modern humans fortuitous by-products afforded by the "spandrel" of greater neocortical capacity [14]?

As mentioned earlier, the relative importance of concerted and mosaic models has mostly been analyzed in mammals [1,11,13-15] with the notable exception of one study on birds [12]. Whether brain structure evolution in the basal, and most diverse vertebrate clade, the Actinopterygii (ray-finned fishes), follows a concerted or mosaic model has yet to be studied even though indirect evidence suggests a role for both mechanisms. For instance, a previous study of a monophyletic clade of Tanganyikan cichlids found that whole brain size correlated significantly with structure volume and that there was variation among structures in the percentage of their variance explained by brain size (32 - 76%) [16]. However, the study was limited to a single clade, included only 7 species and, when analyzing correlations between brain size and structure volume, did not control for phylogenetic effects. Functional comparisons of pallial structures within the vertebrate forebrain suggest that two separate memory systems, a hippocampus based spatial, relational or temporal memory system, and an amygdala based emotional memory system appeared early during evolution [17]. Therefore, the forebrain of vertebrates could share a common, conserved pattern of organization [17]; and hence, there could be conserved developmental patterns spanning across all vertebrate clades. Examples of variation in structure size favoring mosaic evolution can be seen by comparing the medullary lobes of goldfish (*Carassius *sp) and catfish (*Clarias *sp), or the extreme example of the massively enlarged valvula cerebelli and electrosensory lateral line lobe of mormyrids [2]. It has also been suggested that mosaic evolution could be playing a more important role in the evolution of at least certain brain structures of the Actinopterygii [2].

Here we analyzed the relative influence of the mosaic and concerted models of brain structure evolution in the Tanganyikan cichlids, a highly variable clade of ray-finned fishes and the epitome of adaptive radiation [18]. Tanganyikan cichlids are an excellent model for the analysis of rates of phenotypic evolution as they are the most diverse phenotypically, morphologically and behaviorally of the African cichlids and recent morphological analyses have demonstrated the adaptive nature of their radiation [19,20]. Previous analyses have shown that brain size and structure are associated with ecology and behavior [16,21-23], suggesting that, as with other morphological traits, brain size and structure are also highly variable within Tanganyikan cichlids. We combined phylogenetic multivariate allometry analyses [24,25] with recently developed comparative methods that allow for detailed description of rates of phenotypic evolution, even between highly correlated traits, [26,27] to compare evolutionary patterns among major brain structures.

## Results

### Multivariate allometry analyses

The first component (PC1) in the multivariate principal component analysis explained 86% of the total variance in structure volume, the second component (PC2) explained 7% of the variance, or about 50% of the variance not explained by brain size. Finally, although the third component (PC3) only explained about 5% of the total variance, it still contributes to 36% of the variance not explained by changes in brain size, and it is more than four times the fourth component. Two lines of evidence strongly suggest PC1 describes variance in structure volume resulting from differences in brain size. Firstly, all structures increased in size with increasing values of PC1. Secondly, even when controlling for phylogenetic effects, species scores for PC1 were significantly correlated with brain weight (t = -2.92, p = 0.006). Thus, overall changes in brain size explain about 86% of the variance in structure volume. The second and third principal components appear to be related mainly with the size of the dorsal medulla and olfactory bulbs. The dorsal medulla showed a strong positive loading (0.72; Table 1) and the olfactory bulbs a weaker positive loading (0.35; Table 1) on PC2, while all other structures loaded negatively, although the optic tecta, cerebellum and hypothalamus with relatively low loadings (Table 1). These results suggest PC2 mainly describes variation in dorsal medulla volume, which is supported by the fact that the dorsal medulla is the only structure to correlate significantly with PC2 when controlling for phylogeny (t = 3.28, p = 0.002). PC3 appears to separate species based on the size of the olfactory bulbs and dorsal medulla, which interestingly show contrasting patterns: while the olfactory bulbs show a strong positive loading (0.85), the dorsal medulla shows a relatively strong negative loading (-0.51; Table 1) and all other structures show weak negative loadings. Thus, it appears PC2 and PC3 could be associated with sensory specialization, distinguishing between species relying more on gustatory cues and possibly also olfaction from those relying more on visual information.

**Table 1:** Brain structure eigenvector values

**Brain structure**	**PC 1**	**PC 2**	**PC 3**
Olfactory bulbs	-0.376	0.350	0.847

Telencephalon	-0.399	-0.462	-0.086

Optic tecta	-0.382	-0.208	-0.104

Cerebellum	-0.462	-0.287	-0.042

Medulla	-0.431	0.724	-0.513

Hypothalamus	-0.392	-0.123	-0.010

Eigenvector values of the six brain structures on the three main principal component axes (PC1, PC2 and PC3) from a phylogenetic principal components analysis based on the covariance between structures.

Brain structures showed variation in pairwise allometric relationships suggesting relative independence among structures in evolutionary changes in size, even when controlling for phylogenetic effects. The olfactory bulbs presented bivariate allometric coefficients > 1 when compared with all structures, although with some structures the relationship was nearly isometric. On the other hand, the cerebellum presented hypoallometry with all other structures (Table 2). The dorsal medulla also presented hypoallometry with all other structures except for the cerebellum (Table 2). In sum, the allometric coefficients suggest that certain structures, such as the olfactory bulbs, present larger changes in size with changes in other structures, while others, for example the dorsal medulla and cerebellum, change less in size with changes in other structures. These interpretations of the results are supported by the loadings of the individual structures on PC1, the size axis (Table 3). The olfactory bulbs and the dorsal medulla have the lowest loadings of all structures, suggesting a weaker correlation between the volume of these structures and whole brain size.

**Table 2:** Brain structure bivariate allometric coefficients

	**Telencephalon**	**Opctic tecta**	**Cerebellum**	**Medulla**	**Hypothalamus**
Olfactory bulbs	1.06	1.02	1.23	1.15	1.04
Telencephalon		0.96	1.16	1.08	0.98
Optic tecta			1.21	1.13	1.03
Cerebellum				0.93	0.85
Medulla					0.91

Coefficients of the bivariate allometric relationship between brain structures (see Methods for calculation details).

**Table 3:** Brain structure loadings on the first principal component

**Brain structure**	**Loadings on PC1**
Olfactory bulbs	0.86

Telencephalon	0.94

Optic tecta	0.96

Cerebellum	0.98

Medulla	0.88

Hypothalamus	0.98

Loadings of the individual structures on the first principal component of a phylogenetic principal components analysis. These loadings indicate to what extent variance in brain size explains variance in structure volume.

### Rate of evolution analyses

#### Brain size

**Figure 1:**
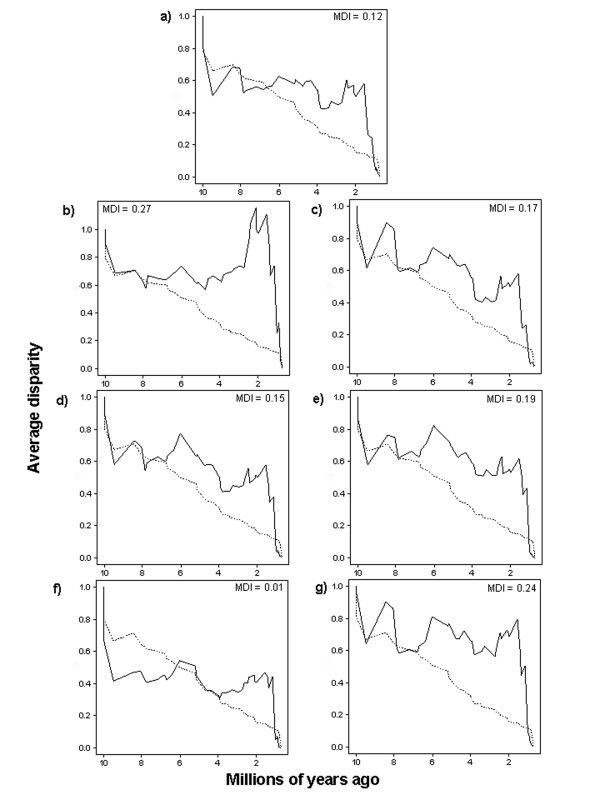
**Morphological disparity through time plots and morphological diversity indices (MDI) for brain weight (a) and the size of six major brain structures: b; olfactory bulbs, c; telencephalon, d; optic tecta, e; cerebellum, f; dorsal medulla and g; hypothalamus**. The bold black line shows the actual morphological disparity of the trait while the broken line shows the median disparity when trait evolution is modeled on the phylogeny following Brownian motion. Time is expressed as millions of years from the present, based on estimated dates for the Tanganyikan cichlid radiation (see Methods).

The rate of evolution of whole brain size did not show a significant departure from the basic Brownian model. The diversity through time plot and the low MDI (0.12) value (Fig 1a), which was lower than that of any brain structure except for the dorsal medulla, both suggest no departure from Brownian evolution of brain size. The result of the morphological disparity analysis is supported by the value of λ, which did not differ significantly from unity suggesting that the evolution of brain weight followed Brownian motion (Table 4). The maximum likelihood value of α did show significant departure from Brownian motion (Table 4). However, although this result contrasts with the MDI value and estimate of λ, the value of alpha is much lower than the estimate for both the olfactory bulbs and the hypothalamus and is closer to the values for the telencephalon, and cerebellum, which did not depart from a Brownian model of evolution. Hence, these results suggest that total brain size evolves in a gradual manner [28].

**Table 4:** Maximum likelihood estimates of the evolutionary parameters

**Brain structure**	**Brownian**	**Lambda**			**Alpha**		
	**Ln Likelihood**	**λ**	**Ln Likelihood**	**P**	***α***	**Ln Likelihood**	**P**

Brain weight	5.69	0.65	7.29	0.07	1.54	7.68	0.05

Olfactory bulbs	-0.36	0.45	4.01	0.003	2.61	3.62	0.01

Telencephalon	2.12	0.80	2.53	0.60	1.57	3.74	0.07

Optic tecta	5.45	0.92	5.53	0.69	1.22	6.56	0.14

Cerebellum	-0.93	0.71	-0.40	0.30	1.42	0.56	0.08

Dorsal medulla	-1.45	0.96	-1.42	0.81	0.40	-1.26	0.53

Hypothalamus	4.79	0.51	6.83	0.04	2.02	7.32	0.02

Maximum likelihood estimators for the λ and α statistics of whole brain weight and six major brain structures. The Ln likelihoods of the null Brownian motion model and those of the two alternative models are shown for comparison. *P *values for the λ and α parameters were determined from likelihood ratio tests against a model with constant rates of evolution (unconstrained Brownian motion).

#### Brain structures

There were notable differences in the rates of evolution of the different brain structures. Both the olfactory bulbs and the hypothalamus presented the highest MDI values of all structures (0.27 and 0.24, respectively), both were 1.25 and 1.18 times larger than the MDI for brain size strongly suggesting that these structures present higher rates of morphological divergence than brain size (Fig 1). The dorsal medulla presented by far the lowest MDI value (0.01) suggesting very gradual rates of divergence. Finally, the telencephalon, optic tecta and cerebellum presented similar MDI values (0.17 and 0.15, and 0.19, respectively) and similar disparity through time plots (Fig 1c, d, e). A pattern worth highlighting is the apparent recent divergence in volume of the olfactory bulbs, as can be seen in the diversity through time plot (Fig 1b). The solid black line, showing morphological divergence in volume of the olfactory bulbs, diverges from the broken line, showing the pattern under Brownian motion, at about 6 MYA and a sharp peak between 4 - 2 MYA. In a similar fashion, although the pattern is less striking, the disparity through time plot for the dorsal medulla (Fig 1f) also suggests that the present interspecific variation in the volume of this structure results from recent evolutionary divergence as shown by the increase in relative disparity observed between 4 and 1 MYA.

The results of the morphological divergence analyses were well supported by the results of the maximum likelihood estimates of λ and α. The only two structures which showed significant departure from Brownian motion were the olfactory bulbs and the hypothalamus (Table 3). Both structures showed clear signals of more rapid rates of evolution than any of the other structures, as was the case when compared with brain size. None of the remaining structures showed any significant departure from Brownian motion (Table 3).

## Discussion

**Figure 2:**
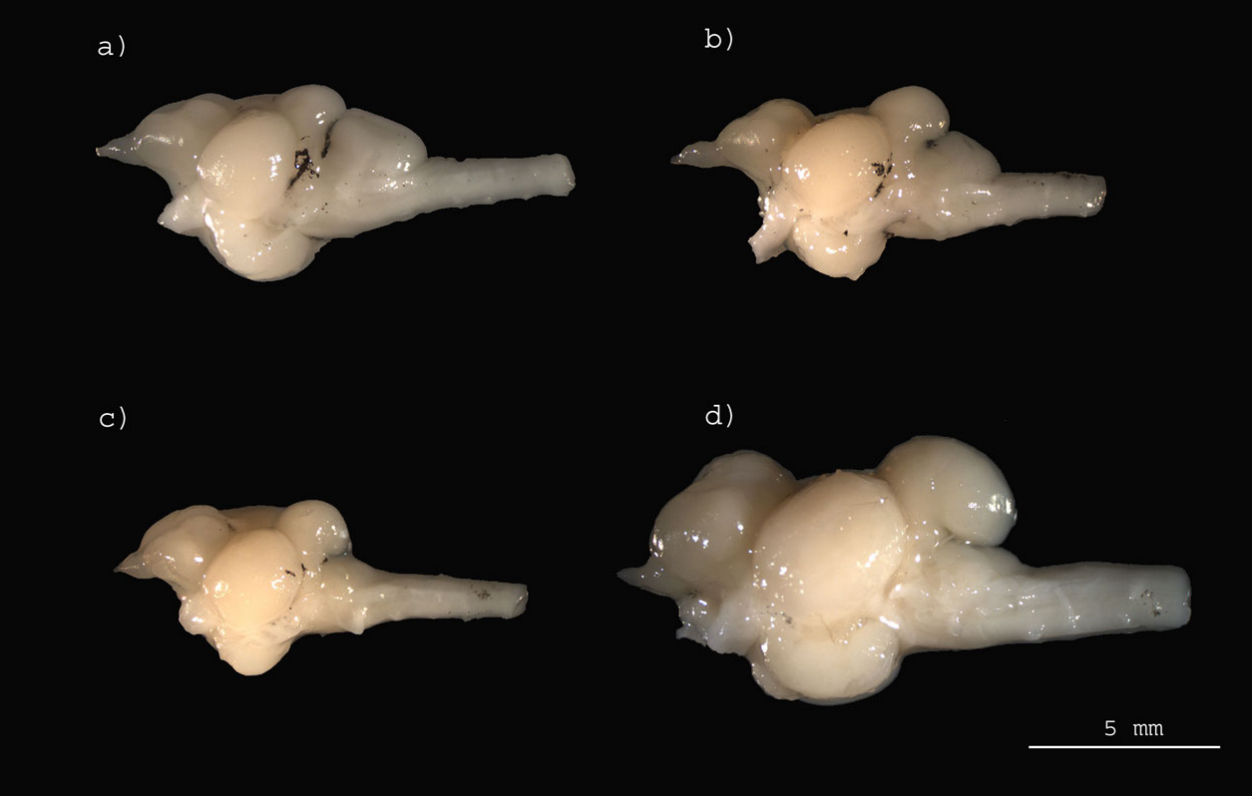
**Brain images of Tanganyikan cichlids in the sagital plane (left side)**: a) *Xenotilapia ochrogenys*, b) *Callochromis pleurospilus*, c)*Triglachromis otostigma*, and d)*Petrochromis orthognathus*.

Overall, our approach based on phylogenetic allometry and rate of phenotypic evolution analyses suggests that brain evolution in Tanganyikan cichlids mainly follows a mosaic model. However, as is the case in both birds and mammals, brain evolution in Tanganyikan cichlids is not purely mosaic and our results also suggest a non-negligible role for concerted evolution [1,3,12]. The results of the phylogenetic multivariate allometry analyses suggest that in Tanganyikan cichlids the variance in brain structure volume explained solely by changes in brain size is much lower than for mammals. While in cichlids differences in brain size accounted for 86% of the variance in structure volume, even when accounting for non-independence of data due to species relatedness, in mammals brain size differences account for 96% of the variance, although in mammals the analysis did not account for phylogenetic effects [13]. This could in part be due to larger size differences in the mammalian sample (range of body weights = 1.86 - 105 000 g) compared to our sample (range of body weights = 0.52 - 135 g), although if brain evolution was strongly influenced by developmental constraints the size range of species included in the sample would not be expected to influence the proportion of variance explained. Therefore, our results suggest that in cichlids brain structures evolve in a more independent fashion from changes in whole brain size as compared with mammals. Further, a previous study has shown that although brain and body size in Tanganyikan cichlids were highly correlated, even when controlling for phylogenetic effects, brain size shows a markedly different pattern of evolution from that of body size [28]. Hence, even highly correlated traits can show distinct patterns of evolution and the results of this study indicate that, at least in cichlids, variance in brain structure volumes is not only explained by the single factor of overall brain size (see Fig 2 for an example of variation in structure volumes across species).

The bivariate allometric coefficients suggest there is relative variation in the allometric relationships between structures, although some structure pairs are nearly isometric. The olfactory bulbs were the most variable brain structure in comparison with all the others, as shown by the fact that this structure showed positive bivariate allometry with all the others and it had the lowest loading on PC1, the size axis, suggesting that its volume is less correlated with whole brain size as compared with other structures. The dorsal medulla, on the other hand, appears to be the least variable of all structures as it presented bivariate coefficients < 1 when compared with all other structures, except for the cerebellum, and presented the second smallest correlation with whole brain size. The results of the allometry analyses are generally supported by the results of the rates of evolution analyses. Only two structures showed consistent and significant departure from a gradual, Brownian motion model of evolution, the first being the olfactory bulbs and the second the hypothalamus (Table 4). Both structures also present evolutionary patterns which contrast with those of the whole brain as with those of other structures, again suggesting that they vary more in size among species than any other structure. The hypothalamus showed varying allometry with other brain structures, ranging from hyperallometry with the cerebellum to slight hypoallometry with the olfactory bulbs. Interestingly, the hypothalamus had the strongest loading on PC1, suggesting its volume is highly correlated with total brain size. Thus, it appears that although the volume of this structure is highly correlated with total brain size, volumetric changes occur more rapidly than in total brain size, as suggested by the high alpha value (Table 4). The dorsal medulla presented the lowest MDI value of all structures, even smaller than MDI for brain size, and it presented the highest λ and smallest α of all structures, suggesting more gradual evolution than other brain structures. The results of the rate of evolution analyses further suggest there has been a recent, rapid divergence in the volume of the olfactory bulbs and hypothalamus among Tanganyikan cichlids. Such rapid divergence is especially notable in the diversity through time plot of the olfactory bulbs (Fig 1b), which presents a recent, high peak in morphological divergence suggesting that interspecific differences in olfactory bulb volume are the result of a recent evolutionary divergence. Although the height of the peak may in part be due to incomplete sampling, which possibly results in overestimation of between species differences [28,29], it is unlikely that the pattern is due solely to incomplete sampling as it is observed only for the olfactory bulbs and not for other structures. Interestingly, the diversity through time plot of the dorsal medulla also suggests that most phenotypic divergence results from recent evolutionary events, even if the pattern is not as striking as for the olfactory bulbs. Such recent divergence suggests potential independent invasion of new niche space, which placed a premium on olfaction, and possibly also taste and other chemosensory abilities, by different lineages during the Tanganyikan radiation. As a whole, these results are in line with what would be predicted under a model of mosaic evolution of brain structure [1,12], since there are clear signals of independent evolution for at least for two of the six brain structures analyzed here. It is worth highlighting that in mammals the olfactory bulb also appears to be the most variable brain structure and it has even been suggested as an exception to the concerted evolution model [13,14].

Furthermore, as would also be predicted under a mosaic model of evolution, our results suggest that specific structures can be the target of selection, rather than all structures changing in size simultaneously, since the second and third principal components appear to separate species based on sensory specialization. The second component mainly describes variation in dorsal medulla size, as shown by the high loading of this structure and by the fact that it is the only structure that correlates significantly with PC2 scores. The third component mainly describes variation in dorsal medulla and olfactory bulb size, and here there appears to be a trade-off between the volume of these structures since the olfactory bulb loads positively and the dorsal medulla negatively on PC3. However, this apparent trade-off must be taken with caution as PC3 describes variation in dorsal medulla size not already accounted for by PC2. In sum, there appears to be a distinction between species relying more on olfaction and chemosensory or taste information on the one hand and species relying more on visual cues on the other, as shown by the fact that while the dorsal medulla and olfactory bulbs load positively on PC2 all other structures show negative loadings. This apparent trade-off between structure sizes could be explained by cognitive demands associated with habitat and diet specialization, both of which have been previously shown to be associated with brain size and structure in Tanganyikan cichlids [16,21-23]. Indeed, the size of the cerebellum is positively correlated with habitat complexity, and the telencephalon showed a similar trend, while olfactory bulb and dorsal medulla sizes are negatively correlated with habitat complexity in a monophyletic clade of Tanganyikan cichlids [16]. In another study analyzing brain structure in cichlids from all three African Great Lakes, areas associated with vision and taste were shown to be associated with differences in feeding habits [22]. Piscivorous taxa, and others feeding on motile prey, had larger optic tecta and cerebellum compared to species feeding on mollusks or plants, while structures relating to taste were well developed in species feeding on benthos over muddy or sandy substrates [22], although the lack of detailed phylogenies at the time precluded any control for phylogenetic effects. Hence, our results are in line with previous findings suggesting adaptive, independent variation in brain structure size in response to specific selection pressures [1,11,12].

Although the variance in structure volume explained by changes in whole brain size is much lower than in mammals, it still represents 86% of all variance in volume. Hence, although brain structure evolution appears to be more flexible in the Tanganyikan cichlids than in mammals, there is still a high proportion of the variance in volume that is explained solely by changes in size [13,14]. Furthermore, except for the olfactory bulbs and the hypothalamus, none of the structures showed evolutionary patterns which differed significantly from those of whole brain size. Further, although pairwise allometry among structures varied in some instances the relationship was nearly isometric (Table 2). Thus, possible developmental constraints, limiting the degree to which structure volumes vary independently of other structures, cannot be ruled out [13,14]. The telencephalon, optic tecta and cerebellum showed similar evolutionary patterns as shown by their highly similar MDI values and by the fact that none differed significantly from Brownian motion (Fig 1c, d). It is interesting to note that in mammals highly interconnected structures have been shown to evolve in a correlated fashion [3], and the telencephalon is highly interconnected with both the optic tecta and cerebellum [30]. Such apparent co-evolution between these structures could result from developmental constraints, however it could also be the result them evolving as a functional system, as has been suggested to be the case for correlated structures in mammals [3]. Hence, high neural connectivity among structures can potentially limit the strength of the signal for mosaic evolution, as some structures would show signals of concerted evolution.

There is some discordance among the results of the rate of evolution analyses for whole brain size. While both the low MDI value and the maximum likelihood estimate of the λ parameter suggest that brain size evolution does not depart from Brownian motion, the maximum likelihood estimate of the α parameter suggests that brain size evolution is possibly better described by an Ornstein-Uhlenbeck model [31,32]. The value of α for brain size (1.54) is much lower than that of both the olfactory bulbs and the hypothalamus (2.61 and 2.02 respectively), both of which showed the highest MDI values and lowest λ values, suggesting that if the rate of brain evolution indeed proceeds faster than under Brownian motion it is still much slower than that of the olfactory bulbs and the hypothalamus [31]. Because the value of α for brain size is only slightly larger than 1, suggesting relatively weak strength of selection (or "pull" towards the optimum), it is possible that the advantage of the OU model versus Brownian motion is the inclusion of a selective optimum rather than evolution of the trait being modeled on a flat landscape. Finally, brain structures cannot evolve completely independently of brain size, as any change in brain structure volume would inevitably influence brain size, thus some degree of correspondence in the evolutionary patterns is to be expected. In any case, non-Brownian evolution of brain size is not supported by either the λ parameter or by the low MDI value.

## Conclusion

In sum, our results provide robust evidence for an important role of mosaic evolution of the cichlid brain, as shown by the differing bivariate allometries among structures, the different rates of evolution of brain structures and the apparent trade-off between primary sensory structures. These results are in line with previously described associations between structure size and species' ecology in Tanganyikan cichlids [16,22]. Furthermore, brain developmental patterns of other ray-finned fishes also suggest a predominant role for mosaic evolution. The early development of the neural tube in zebrafish (*Danio rerio*) has been shown to take place simultaneously in several independent centers. Differentiated neurons appear in small, isolated, bilaterally symmetrical clusters which give rise to specific structures, and the increase in differentiated neuron number occurs at different rates during development in the different clusters [33]. Thus, as a result of such a developmental pattern, it is possible that by increasing or reducing the rate of accumulation of differentiated neurons at specific, independent clusters, final structure volume could be modified relatively independently from other structures. Furthermore, differential growth patterns of brain structures from the juvenile to adult stages could accentuate initial differences. Comparison of brain growth patterns in four cyprinid fish species showed that there is much greater structural similarity among brains of juveniles of different species than among brains of adult specimens and species differences in brain morphology apparently become increasingly pronounced by lifelong allometric growth [34]. As would be predicted under a mosaic model of evolution, differential growth of structures during the late-larval and juvenile periods has been suggested to produce distinct types of brain organization in cyprinids, probably related to different sensory capabilities [1,30,34]. A combination of initial developmental differences and lifelong allometric growth of structures could potentially also explain the variation in the bivariate allometric relationships between brain structures in Tanganyikan cichlids and the next step is to analyze potential ecological, behavioral and sexually selected correlates of brain structures.

Our results also highlighted some similarities in the evolution of mammal and cichlid brains. As in mammals the most variable structure in Tanganyikan cichlids was the olfactory bulbs. Moreover, highly interconnected structures, the telencephalon, optic tecta and cerebellum, showed signals of correlated evolution in the Tanganyikan cichlids, and in mammals it has been suggested that patterns of correlated evolution can be to a great extent predicted by anatomical connectivity [3]. Previous studies have suggested there are several homologous structures with conserved functions between mammalian and teleost telencephalons, notably the pallial structures [17,35-37]. Our results thus add to the evidence for a common, conserved developmental pattern, and possibly basic organization, across vertebrates.

## Methods

### Data

We obtained volumetric measures of brain structures for 43 Tanganyikan cichlid species [see Additional file 1]. Our sample included most Tanganyikan species for which detailed phylogenetic information is available, and provides a representative sample of natural variation in the lake, including 7 out of the 12 tribes into which Tanganyikan cichlids have been grouped [38].

Brains were collected from wild caught, sexually mature individuals. Fish were first deeply anesthetized with benzocaine and then the head was severed and preserved in 4% paraformaldehyde in a phosphate buffer for tissue fixation and preservation. Whole brain weight (± 0.001 g) was obtained from dissected brains following fixation. All weights were obtained using a Precisa 125A electronic scale (precision = 10^-5 ^g; Precisa Instruments AG, Switzerland). All cranial nerves, optic nerves and meningeal membranes were removed and the brain was severed from the spinal cord 2 - 3 mm posterior of the dorsal medulla. The number of days samples spent in paraformaldehyde prior to dissection had no effect on brain weight even when controlling for body weight (t = -0.83, p = 0.41, n = 194). Intraspecific sample sizes = 3 - 7 individuals, except for one species for which we had two samples, and two species for which we only had one sample [see Additional file 1].

All dissections, digital images and measurements were performed by the same person (AG-V). All were done blindly since specimens were identified by number and not species name. Digital images of the dorsal, ventral, left and right sides of the brain were taken through a dissection microscope (Leica MZFLIII), using a digital camera (Leica DFC 490 and Firecam v. 3.1 software). For each image the brain was carefully placed on a Petri dish with 0.9% agar, which was solid but would yield to brains and allow for them to be placed in such a manner to ensure that the view of the brain being photographed was horizontal and both sides were symmetrical. For paired structures, both were measured and the volume was the sum of the two structures. We followed the procedure of Pollen et al. [16] to measure length, width and height of six key-structures: olfactory bulbs, telencephalon, optic tecta, cerebellum, hypothalamus and dorsal medulla. The volume of each structure was quantified according to the ellipsoid model: V = (L × W × H) π/6, which provides consistent estimates of the volume of brain structures in Taganyikan cichlids [16,21-23] even when compared to volumes obtained from sectioning on a microtome followed by staining [16]. Although the model sometimes over-estimates structure volume as compared to estimates from sectioning, part of the overestimation is due to the shrinkage that occurs with the normal drying and dehydration process during staining [16]. Because our approach is based on interspecific patterns of phenotypic evolution, such slight overestimation would bias our analyses if the overestimation differs notably between species. However, based on the limited amount of available data for comparison of volume estimates between the ellipsoid model and volume estimates from sections [16], the overestimation of structure volume by the ellipsoid model appears similar across species. To estimate repeatability the volume of all structures was measured twice on one randomly picked specimen from each of the 43 species. In all cases the correlation coefficient between repeated measures for all structures was high, r > 0.98. To verify that intraspecific variability was similar among structures, we compared the species-specific standard errors across the 6 structures. There was no significant difference between structures (F = 1.91, p = 0.09, df = 5, 257; none of the post-hoc analyses were significant: range of p values = 0.22 - 1.00), suggesting that there is no systematic bias. All data was log_10 _transformed and because some of the measures were smaller than 1, we multiplied all data by 1000 prior to log transformation [39].

In brief, the main functions of these six brain structures in the vertebrate brain are as follows: the olfactory bulbs receive olfactory signals, which are then relayed directly to the telencephalon [2,4,40]. The telencephalon forms the cognitive center of the vertebrate brain that processes all sensory information and also plays important roles in directing active movements as well as in learning and memory [2,4,17,40]. A key function of the optic tecta, especially in fishes, is to receive visual information, which is then transferred to the telencephalon via the preglomerular zone [41,42], the optic tecta also receive other sensory modalities in addition to retinal inputs and appear to play a role in control of movement [2,4,40,43]. The cerebellum is the center for motor control and coordinates muscle activity, movements and balance [2,4,40], although the cerebellum may also be involved in learning and memory [44]. The hypothalamus is functionally connected to the pituitary gland and controls many basic bodily functions such as reproduction and growth as well as motivation and the autonomic nervous system [2,4,40]. Finally, the dorsal medulla receives lateral line, taste projections, and auditory information, with rostral components processing mechanosensory lateral line stimuli and more caudal and medial aspects relating to taste [2,4,40,45].

### Phylogenetic multivariate allometry analyses

First, we analyzed allometric relationships among individual structures using co-variance based principal component analysis in R taking into account the phylogenetic relationships among species. Principal components analysis assumes that the sample consists of independent data points, an assumption that is violated by data from species sharing varying degrees of plylogenetic relatedness [24,39]. Thus, the analyses were undertaken using species specific values (rather than using evolutionary differences between species, i. e. independent contrasts) and calculated based on the evolutionary variance-covariance matrix among species given by the phylogenetic relationships among species [24]. Because some of our traits showed significant departure from a Brownian motion model of evolution (see Results), we did not compute the evolutionary variance-covariance matrix based on a Brownian motion model of evolution but rather on a matrix corrected by the lambda parameter (see below; [24,27,46]). For this correction we used the average value of the maximum likelihood estimates of lambda for the six brain structures, each of which was estimated for each structure individually (see below). Although we acknowledge that this estimate possibly differs from a multivariate estimate for the data matrix as a whole, it is as yet not possible to estimate such parameters for multivariate data sets.

The ratio of PC1 coefficients for any pair of variables corresponds to the variables' bivariate allometric coefficient [25]. Under a concerted evolution model the bivariate allometry among structures is not predicted to depart markedly from isometry. Under a mosaic model bivariate allometry is predicted to depart from isometry as structures grow or shrink independently of changes in size of other structures. We used the loadings of the individual structures on the first principal component as an estimate of their correlation with variation in whole brain size. Loadings are the correlation between the phenotypic data and the component scores on the transformed space. Use of multivariate allometric analyses has the advantage of avoiding problems with adequate controls for size when analyzing intercorrelations between structures as well as problems of multicolinearity in multiple regression analyses, as structure volumes are often highly correlated [39,47-49].

### Phylogenetic comparative analyses

For the comparative analyses we used a molecular phylogeny reconstructed using mitochondrial sequences downloaded from Genbank (for details on phylogeny reconstruction see [28]). The phylogeny has previously been shown to provide an accurate representation of speciation patterns for Tanganyikan cichlids [28]. All correlations controlling for phylogenetic effects were done using phylogenetic generalized least squares [50] in R, using the package ape [51]. To analyze patterns of evolution we applied two complementary methods: the morphological diversity index [26] and a maximum likelihood estimate of the lambda (λ) and alpha (α) parameters [27]. Both methods test for departure from a null Brownian motion model of evolution, where phenotypic divergence accumulates with time in a stochastic manner, but differ in the approach used to test for this departure, the additional information they provide on rates of character evolution as well as in their limitations [28].

To examine the patterns of morphological evolution, we calculated disparity through time plots [26] for all brain structures and brain weight, using the package GEIGER [52] in R following Harmon et al. [26]. Morphological differences (disparity) were calculated from average pair wise Euclidean distances between species. Disparity through time was calculated as the average relative disparity of each subclade by dividing the average disparity of all subclades whose ancestral lineages were present at that time by the average disparity of the clade as a whole, and repeating this at each divergence event (i.e., each node) moving up the phylogeny from root to tip. A null hypothesis was constructed by simulating morphological divergence of each trait along the phylogeny under an unconstrained Brownian motion model. The morphological diversity index (MDI) was calculated as the sum of the areas between the curve describing the morphological disparity of the trait and the curve describing the disparity under the null hypothesis of Brownian motion. Areas in which observed values were above expected were assigned positive values, whereas those below expected were assigned negative values. The MDI thus describes how morphological diversity is partitioned within the clade: values above 0 indicate that most morphological disparity is distributed within clades, suggesting recent phenotypic divergence, negative values suggest disparity is distributed among clades, suggesting early divergence, while values near 0 indicate evolution has followed Brownian motion. For ease of interpretation, in the disparity through time plots we present the time scale as million of years to the present, using 10 million years ago as an estimate for the origin of the Tanganyikan cichlid radiation [38]. The advantage of the MDI is that it avoids reconstruction of ancestral states and it provides a graphical representation, as well as a numerical index (MDI), of the pattern of morphological divergence along the phylogeny. A limitation of the MDI is that there is no statistical test of departure from the null Brownian motion model of evolution.

Second, we calculated maximum likelihood values for the λ parameter which tests whether traits evolve according to the null Brownian motion model (λ = 1)[27] and for the α parameter, which is based on an Ornstein-Uhlenbeck (OU) process and estimates the strength of selection acting on the trait [31,32], using the package GEIGER [52]. The OU model is the simplest mathematical expression for an evolutionary model incorporating selection and it differs from a Brownian model in that it possesses a selective optimum [31]. It is important to note, however, that it also includes Brownian motion as a special case [31]. The OU model has two terms:



the first term describes change in character × over the course of a small increment in time, the second term is random variation accumulating with time, or in other words a Brownian process [31]. The parameter α describes the strength of selection, the higher the value of α, the stronger the selective regime; θ is the value of the selective optimum [31]. Under an OU model, the rate of phenotypic change along the branches of a phylogeny depends on two things, the distance between the actual trait value and the value of the selective optimum, and the strength of the "pull" towards the selective optimum, given by the value of α. Hence, the higher the value of α, the stronger the "pull" towards the selective optimum, in other words as α increases divergence will accumulate increasingly rapidly along the branches of the tree as compared to a basic Brownian process [31]. It can also be seen that as α becomes increasingly small the OU model will eventually reduce to a Brownian process and as α tends towards 1 the process will reduce to a model with one selective optimum but with no accelerated accumulation of divergence [31]. Because the OU model is to a certain point an extension of the Brownian model, traits presenting very small departures from Brownian motion may yield higher likelihood values when their evolution is modeled by an OU process. When comparing between models the importance lies in the value of α and thus the strength of the selection regime. We refrained from estimating maximum values of δ because it has been suggested that this parameter, which is similar to Grafen's ρ, could be biased [29].

The advantage of using GEIGER to estimate the values of the evolutionary parameters is that it can incorporate an estimate of intraspecific variance into the analyses, in this case the standard error of volumes of brain structures and brain weight for each species (in the few cases where a single sample was available for a species the error was set to 0). A p-value was obtained by comparing the models with the λ and α parameters to a null model of unconstrained Brownian motion with the log-likelihood statistic. By combining three analyses of the rates of evolution, a result suggesting departure from Brownian motion will be robust if all three results present patterns which are consistent with a departure from Brownian motion [28].

Under a concerted evolution model we predicted that all brain structures would present similar patterns of evolution and that these would follow those of whole brain size, since structures are predicted to increase in size as a response of changes in whole brain size [13,14]. On the other hand, under a mosaic evolution model we would expect divergence in patterns of evolution between whole brain size and individual brain structures, and especially among structures, as these are predicted to vary in size independently [1].

## Authors' contributions

All authors have read and approved the final manuscript. AG-V participated in the design of the study, conducted brain dissections and data collection, carried out the analyses and drafted the manuscript. SW assisted with brain dissections and critically revised the manuscript. NK conceived of the study, obtained funds, participated in the design and coordination of the study and helped to draft the manuscript.

## Supplementary Material

Additional file 1 **List of sampled species and sample sizes per species**. Table presenting the list of sampled species and the sample size per species. Click here for file
