# Supplementary material for: Brain structure evolution in a basal vertebrate clade: evidence from phylogenetic comparative analysis of cichlid fishes
Source: BMC Evol Biol. 2009 Sep 21;9:238. doi: 10.1186/1471-2148-9-238 (PMC2755010; doi:10.1186/1471-2148-9-238)
Supplement: Additional file 1 — List of sampled species and sample sizes per species. Table presenting the list of sampled species and the sample size per species. [file 1471-2148-9-238-S1.DOC]

Supplementary material

List of sampled species and number of sampled individuals per species.

| Species | Sample size |
| --- | --- |
| Altolamprologus compressiceps | 6 |
| Aulonocranus dewindti | 6 |
| Benthochromis tricoti | 2 |
| Callochromis melanostigma | 6 |
| Callochromis pleurospilus | 4 |
| Ctenochromis horei | 3 |
| Cyathopharynx furcifer | 4 |
| Cyphotilapia frontosa | 5 |
| Cyprichromis microlepidotus | 5 |
| Cyprichromis leptosoma | 8 |
| Enantiopus melanogenys | 4 |
| Eretmodus cyanostictus | 4 |
| Gnathochromis permaxillaris | 4 |
| Greenwoodochromis christyi | 3 |
| Haplotaxodon microlepis | 6 |
| Julidochromis ornatus | 6 |
| Julidochromis transcriptus | 6 |
| Julidochromis marlieri | 6 |
| Julidochromis regani | 6 |
| Lamprologus callipterus | 4 |
| Lepidiolamprologus nkambae | 6 |
| Limnochromis auritus | 6 |
| Limnotilapia dardennii | 6 |
| Neolamprologus brevis | 3 |
| Neolamprologus brichardi | 7 |
| Neolamprologus tetracanthus | 1 |
| Neolamprologus tretocephalus | 7 |
| Ophthalmotilapia boops | 3 |
| Ophthalmotilapia nasuta | 4 |
| Ophthalmotilapia ventralis | 4 |
| Paracyprichromis breini | 6 |
| Petrochromis famula | 1 |
| Petrochromis orthognathus | 4 |
| Simochromis babaulti | 5 |
| Spathodus marlieri | 6 |
| Spathodus erythrodon | 4 |
| Tanganicodus irsacae | 4 |
| Triglachromis otostigma | 6 |
| Tropheus brichardi | 4 |
| Tropheus moorii | 4 |
| Xenotilapia ochrogenys | 6 |
| Xenotilapia spilopterus | 6 |
| Xenotilapia flavipinnis | 4 |
